# 145. SARS-CoV-2 (COVID-19) Testing Experience within a Military Treatment Facility

**DOI:** 10.1093/ofid/ofab466.145

**Published:** 2021-12-04

**Authors:** Sara Robinson, Wesley R Campbell, Yuliya Johnson, Michael Backlund, Daniel Brooks

**Affiliations:** 1 WRNMMC, Bethesda, Maryland; 2 Walter Reed National Military Medical Center, Kensington, MD

## Abstract

**Background:**

The Walter Reed National Military Medical Center (WRNMMC) established a consolidated COVID-19 screening area (CSA) beginning in March 2020 to provide beneficiary and staff testing via a drive-through site. Testing was available to all patients and WRNMMC staff regardless of beneficiary status. Presented is a descriptive analysis of our testing operations and positivity rates within a closed medical system from March 2020 to April 2021.

**Methods:**

For quality and process improvement, we compiled daily testing logs from March 2020 to April 2021 from the CSA. These logs included patient demographics, reason for testing, test result, testing platform, and occupational status at the hospital. We determined positivity rates in various subgroups – asymptomatic, symptomatic, pre-operative, in order to track testing use and access. Additionally, we compared the overall positivity rate to the surrounding civilian community by pulling data from the Maryland Department of Health’s COVID database.

**Results:**

Over the course of nearly 14 months of testing availability, 34,694 beneficiaries were screened with 41,582 individual tests. After May 2020, the monthly overall positivity rate varied from 1.99% to 11.92%, peaking in December 2020 (with high rates in November 2020, 7.52% and January 2021, 9.53%), correlating with or exceeding elevated positivity rates in Montgomery County (November 2020: 4.91%; December 2020: 6.48%; January 2021: 6.51%). When examining only symptomatic individuals, the positivity rate is notably much higher, with monthly rates varying from 6.40% to 21.10%, with a similar peak in December 2020. After full implementation of pre-operative screening for procedures with aerosolization potential in June 2020, the range of positivity rates was 0.28%-1.66%. Since vaccination for COVID-19 became widely available beginning in Feb 2021, the preoperative positivity rate has remained below 0.85%.

**Conclusion:**

Our institutional experience is unique in its ability to offer universal access to COVID-19 testing for beneficiaries and staff of the DoD under direction of the ID service. Our process serves as a model for public and occupational health response, and may guide lab resource and real-time staffing management in support of COVID-19 diagnostics at a medical center.

**Disclosures:**

**All Authors**: No reported disclosures

